# Modulation of Energy Metabolism Is Important for Low-Oxygen Stress Adaptation in Brassicaceae Species

**DOI:** 10.3390/ijms21051787

**Published:** 2020-03-05

**Authors:** Ji-Hye Hwang, Si-in Yu, Byeong-ha Lee, Dong-Hee Lee

**Affiliations:** 1 Graduate Department of Life and Pharmaceutical Sciences and the Center for Cell Signaling & Drug Discovery Research, Ewha Womans University, Seoul 03760, Korea; hwangjihye0012@gmail.com; 2 Department of Life Science, Sogang University, Seoul 04107, Korea; siin0311@sogang.ac.kr

**Keywords:** low oxygen, hypoxia, energy metabolism, Brassicaceae, gene set enrichment analysis (GSEA)

## Abstract

Low-oxygen stress, mainly caused by soil flooding, is a serious abiotic stress affecting crop productivity worldwide. To understand the mechanisms of low-oxygen stress responses and adaptation of plants, we characterized and compared low-oxygen responses in six species with different accessions of the Brassicaceae family. Based on the growth and survival responses to submergence or low-oxygen condition, these accessions could be divided into three groups: (i) Highly tolerant species (*Rorippa islandica* and *Arabis stelleri*); (ii) moderately tolerant species (*Arabidopsis thaliana* [*esk-1*, Ler, Ws and Col-0 ecotype]); and (iii) intolerant species (*Thlaspi arvense*, *Thellungiella salsuginea* [Shandong and Yukon ecotype], and *Thellungiella parvula*). Gene expression profiling using Operon Arabidopsis microarray was carried out with RNA from roots of *A. thaliana* (Col-0), *A. stelleri*, *R. islandica*, and *T. salsuginea* (Shandong) treated with low-oxygen stress (0.1% O_2_/99.9% N_2_) for 0, 1, 3, 8, 24, and 72 h. We performed a comparative analysis of the gene expression profiles using the gene set enrichment analysis (GSEA) method. Our comparative analysis suggested that under low-oxygen stress each species distinctively reconfigures the energy metabolic pathways including sucrose–starch metabolism, glycolysis, fermentation and nitrogen metabolism, tricarboxylic acid flow, and fatty acid degradation via beta oxidation and glyoxylate cycle. In *A. thaliana*, a moderately tolerant species, the dynamical reconfiguration of energy metabolisms occurred in the early time points of low-oxygen treatment, but the energy reconfiguration in the late time points was not as dynamic as in the early time points. Highly tolerant *A. stelleri* appeared to have high photosynthesis capacity that could produce more O_2_ and in turn additional ATP energy to cope with energy depletion caused by low-oxygen stress. *R. islandica* seemed to retain some ATP energy produced by anaerobic energy metabolism during a prolonged period of low-oxygen conditions. Intolerant *T. salsuginea* did not show significant changes in the expression of genes involved in anaerobic energy metabolisms. These results indicate that plants developed different energy metabolisms to cope with the energy crisis caused by low-oxygen stress.

## 1. Introduction

Plant growth and development is highly dependent on a variety of environmental conditions such as temperature, light, water availability, and soil conditions. Among these environmental restrictions, flooding is one of the most severe factors that reduce the productivity of crops. Oxygen deficiency (or hypoxia) is the main stress during soil flooding and leads to a decrease in cellular energy charge, cytoplasmic pH, and accumulation of toxic end products from anaerobic respiration and reactive oxygen species (ROS) during recovery [1,2]. Plants have developed adaptation mechanisms at the morphological, anatomical, and molecular levels to enhance their survivability in low-oxygen conditions. 

Low oxygen causes a dramatic change in gene expression and protein synthesis in plants. Oxygen deficiency in plant cells leads to the enhancement of metabolism such as sucrose catabolism, glycolysis, and fermentation pathways, which are important for energy conservation [3,4]. Hypoxia-induced genes and proteins include signal transduction components and transcription factors, as well as those involved in nitrogen metabolism, ethylene biosynthesis, and cell wall loosening [5,6,7]. The mechanisms to combat low-oxygen stress have been intensively studied over the past years, and these studies mainly used *A. thaliana* and rice as a model system [2,8,9,10,11,12,13,14]. Recently, low-oxygen stress responses were also studied in other species such as Rorippa and Rumex, which are known as tolerance species to submergence and flooding [13,15,16]. The comparative analysis of various species with different low-oxygen stress tolerance levels could overcome the narrow model plant-based knowledge and provide a useful basis to understand the conserved and unique mechanisms by which species adapt to low oxygen. 

Species closely related to Arabidopsis, such as *Arabis stelleri*, *Rorippa islandica*, *Thlaspi arvense*, *Thellungiella salsuginea*, and *Thellungiella parvula* in the Brassicaceae (or Cruciferae) family have been studied for adaptive responses to various abiotic stresses [17,18,19,20,21,22,23,24]. The morphology and growth rate of species closely related to Arabidopsis look very different from each other (Supplementary Figure S1A). In the phylogenetic relationship of these species based on internal transcribed spacer (ITS) sequence, *A. thaliana* was more closely related to *A. stelleri* and *R. islandica* than three species of *Thellungiella* genus and *T. arvense* (Supplementary Figure S1B).

The perennial herb *A. stelleri* (or rock cress) is found in seaside sand or between rocks. Recent studies have shown that *A. stelleri* var *japonica* is stronger in osmotic stress tolerance than *A. thaliana* [17]. *R. islandica* occurs in high mountain sites and inhabits on the banks of mountain streams and shores of lakes [25]. It has been reported that Rorippa species such as *Rorippa sylvestris* and *Rorippa amphibia* could tolerate deep submergence [15]. *T. arvense* is a winter annual cruciferous weed, commonly known as stinkweed, which can survive extreme winter temperatures in Canadian Prairies [26]. With such features in growth habitats, *T. arvense* has recently been recognized as a model plant for freezing tolerance [18]. *T. salsuginea* (salt cress; also called *T. halophila*) is native to harsh environments. The Shandong and Yukon ecotypes of *T. salsuginea* have been proposed as new model plants for research on abiotic stress tolerance [19,20,21,22]. The Shandong ecotype grows in high-salinity coastal areas in eastern China, and has been proposed to be an appropriate relative of Arabidopsis for studies of salinity tolerance mechanisms [19,27]. On the other hand, the Yukon ecotype was isolated in the Takhini Salt Flats near Whitehorse in the Yukon Territories, Canada, a subarctic and semiarid region [28] characterized by multiple simultaneous abiotic stresses, including cold, drought, and high salinity [21]. Another species, *T. parvula* is as tolerant as *T. salsuginea* under salt and drought stress, but has distinct morphological features [23,29]. Despite numerous abiotic stress-related studies of species closely related to Arabidopsis, the low-oxygen stress responses of these species have yet to be explored, expect for Rorippa species. *Rorippa sylvestris* and *Rorippa amphibia* were studied for submergence response including plant survival, changes in carbohydrate and metabolite concentrations, and transcriptome [15]. 

In this study, we compared the physiological and transcriptional responses to low-oxygen stress with Arabidopsis and its related species to gain comprehensive insights into the low-oxygen responses of the species. We first compared the physiological responses of a total of six species with different accessions, including three ecotypes of *A. thaliana* (Columbia (Col-0), Landsberg erecta (Ler), and Wassilewskija (Ws), freezing-tolerant Arabidopsis *esk-1* mutant (Coumbia ecotype)), *A. stelleri*, *R. islandica*, *T. arvense*, Shandong and Yukon ecotypes of *T. salsuginea* and *T. parvula* under low-oxygen stress. Based on these physiological responses, we selected four species and considered *A. thaliana* (Col-0) as a moderately tolerant species, *A. stelleri* and *R. islandica* as highly tolerant species, and Shandong ecotype of *T. salsuginea* as intolerant species under low-oxygen stress. With these four species, gene expression profiling using an Operon Arabidopsis microarray was carried out at various time points over 72 h, and the gene expression profiles were comparatively analyzed using gene set enrichment analysis (GSEA).

## 2. Results

### 2.1. Physiological Response to Low-Oxygen Stress in Species Closely Related to Arabidopsis

In order to compare the levels of tolerances to low-oxygen stress in six species with different accessions, *A. thaliana* and closely related species at a similar developmental stage before bolting were treated with submergence in water or low-oxygen gas treatment (0.1% O_2_/99.9% N_2_), each of which causes hypoxia conditions. 

**Figure 1:**
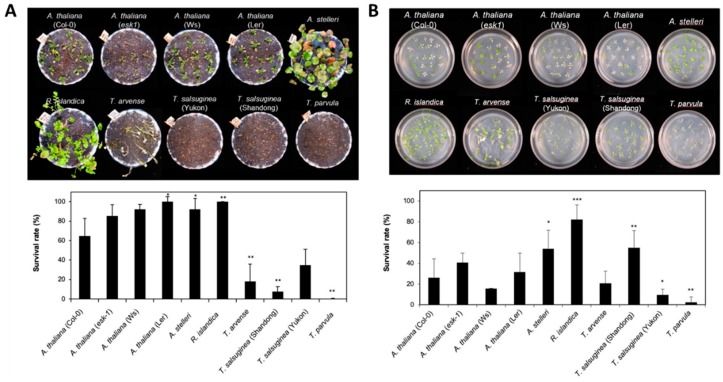
Comparison of tolerance to low-oxygen stress of *A. thaliana* and closely related species. (**A**) Morphology of six species with different accessions after submergence treatment. Three-week-old Arabidopsis and four-week-old closely related species, similar in development stage (immediately before bolting), in pots were entirely submerged for 7 days. Photographs were taken after recovery for 7 days. The survival rate was measured after 7 days of recovery. One pot contained more than 15 plants, and each experiment was repeated three times. (**B**) Morphology of six species with different accessions after low-oxygen gas treatment. Two-week-old Arabidopsis and three-week-old closely related species on agar plates, with three to four leaves at a similar development stage, were exposed to a low oxygen gas mixture (0.1% O_2_/99.9% N_2_) for 5 days. Photographs were taken after 3 days of recovery. The survival rate was measured after 3 days of recovery. One plate contained more than 20 plants, and each experiment was repeated three times. Bars represent the mean (three times) ±standard deviation. In the comparison between the survival rate of A. thaliana and each species, one asterisk (*) indicates *p* < 0.05; double asterisks (**), *p* < 0.01; and triple asterisks (***), *p* < 0.001.

In most species after submergence treatment, morphological changes such as shoot elongation and leaf chlorosis were observed, and their growth was inhibited. The damage levels (i.e., hypoxia tolerance) varied in the six species with different accessions (Figure 1A). *T. arvense* and three species of *Thellungiella* genus (Yukon, Shandong, and *T. parvula*) were most severely affected by this treatment (Figure 1A). After 7 days of recovery under normal growth conditions, *A. thaliana*, *R. islandica,* and *A. stelleri* showed relatively higher survival rates over 67%, whereas *T. arvence* and three species of *Thellungiella* genus displayed lower survival rates ranging from 0% to 35% (Figure 1A).

The low-oxygen gas treatment affected the six species with different accessions differently, but similarly to submergence treatment (Figure 1B). *R. islandica* and *A. stelleri* were more lightly damaged by the low-oxygen gas treatment compared with other accessions; the survival rate of *R. islandica* was higher than 80%, while *T. parvula* had the lowest survival rate at 3% (Figure 1B).

Based on the growth and survival responses to submergence or low-oxygen gas treatment, the six species with different accessions could be divided into three groups: (i) highly tolerant species such as *R. islandica* and *A. stelleri*, (ii) moderately tolerant species such as *A. thaliana* (*esk-1*, Ler, Ws and Col-0), and (iii) intolerant species such as *T. arvense*, *T. salsuginea* (Shandong and Yukon), and *T. parvula* (Table 1).

### 2.2. Expression Profile Analysis of the Low-Oxygen Response in A. thaliana, A. stelleri, R. islandica, and T. salsuginea

To understand the differences in low-oxygen tolerance at the molecular level, we carried out gene expression profiling of *A. thaliana* (Col-0; moderately tolerant species), *A. stelleri*, and *R. islandica* (highly tolerant species), and Shandong ecotype of *T. salsuginea* (low-oxygen sensitive species). The microarray experiments were performed using RNA from root samples taken at various time points of 0, 1, 3, 8, 24, and 72 h after transfer to a low-oxygen condition. As these species are closely related to Arabidopsis, the Operon Arabidopsis Version 3.0 microarray was used with hybridizations after dye swap and untreated *A. thaliana* as a common reference (Supplementary Table S1). The reproducibility values between the dye swap replicates were shown in the range of 0.75 to 0.99.

The correlation coefficients (R^2^) of statistical relationships between microarray signal intensities of *A. thaliana* and each of the closely related species were high; 0.79 of *A. stelleri*, 0.94 of *R. islandica*, and 0.75 of *T. salsuginea* in comparison with *A. thaliana*. The average of hybridization signal intensities was also similar between *A. thaliana* and each of the closely related species. These results suggested that the Arabidopsis microarray can be used for gene expression analysis of closely related species. All transcripts of *A. thaliana* (Col-0), *A. stelleri*, *R. islandica*, and *T. salsuginea* (*Shandong*) are shown in Supplementary Table S2.

To verify the microarray results of close relatives, semi-quantitative RT-PCR assays were carried out (Supplementary Figure S2). A total of 10 hypoxia-responsive genes were selected from the published data (Supplementary Table S3; [6,13]) and used for the verification assay. Because of a lack of sequence information in close relatives, Arabidopsis sequence-based primers could not amplify all ten gene transcripts in close relatives. However, semi-quantitative RT-PCR results of these amplified genes showed that these genes were highly expressed in closely related species under low-oxygen treatment (Supplementary Table S3). The genes confirmed in close relatives were also shown to be inducible in Arabidopsis (Supplementary Figure S2). Unfortunately, no primers for these genes worked for *T. salsuginea* transcripts, which indicated a larger variation of the gDNA of this species from Arabidopsis one. Therefore, only for microarray confirmation, the primers of some known *T. salsuginea* cDNA sequences (i.e., *ABI1*, *RPS25A*, and *CPK9*) and one Arabidopsis-specific gene (*LACS4*) that was randomly picked and shown to work were used for RT-PCR. When the tubulin β2 (TUB2)- or actin-normalized RT-PCR intensity values were compared with the gene expression levels detected in microarray data, there were relatively high correlations (0.5 < R^2^ < 0.9) (Supplementary Figure S2), indicating that our microarray data were reliable.

**Figure 2:**
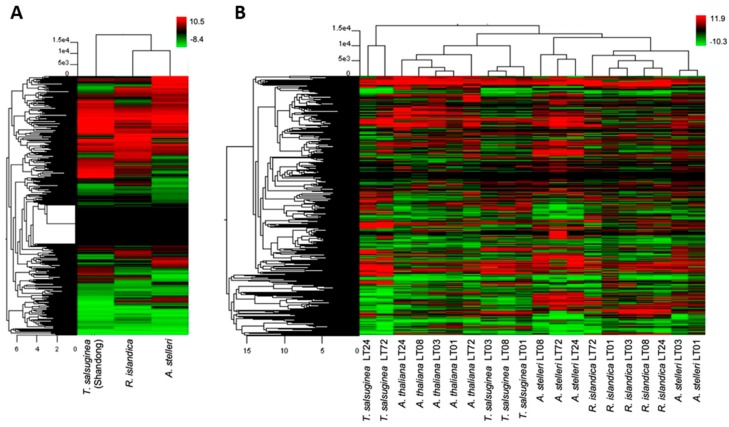
Hierarchical cluster of gene expression patterns in the four species. Gene expression patterns of *A. thaliana*, *R. islandica*, *A. stelleri*, and *T. salsuginea* (*Shandong*) under normal conditions (**A**) or low-oxygen stress (**B**) were clustered by Euclidean distance. Logarithmic scales indicating the color assigned to each fold change are shown to the right of cluster. LT: low-oxygen treatment.

We performed hierarchical clustering to compare global changes in the gene expression profiles of *A. thaliana* and close relatives. In normal growth conditions, hierarchical clustering with Arabidopsis as a reference grouped *R. islandica* and *A. stelleri* closely together (Figure 2A). This clustering revealed that under normal growth conditions, the gene expression patterns of both highly hypoxia-tolerant *R. islandica* and *A. stelleri* were similar to each other and the expression patterns of hypoxia-sensitive *T. salsuginea* were different from those of these two species. In low-oxygen stress, hierarchical clustering showed that the gene expression data of *A. thaliana* and close relatives were largely divided into three groups (Figure 2B). With respect to the most strongly regulated genes, the low-oxygen responses of *R. islandica* were similar to those of *A. stelleri*, while those of moderately hypoxia-tolerant *A. thaliana* were similar to the responses of low-oxygen sensitive *T. salsuginea* at time points of 1, 3, and 8 h. By contrast, the late responses of *T. salsuginea* at time points of 24 and 72 h were different from those of the aforementioned. These observations suggested that the differences in degree of low-oxygen stress tolerance were reflected in the gene expression patterns of each species under low-oxygen stress.

### 2.3. Pathway Profiles Based on Gene-Set Enrichment Analysis

**Figure 3:**
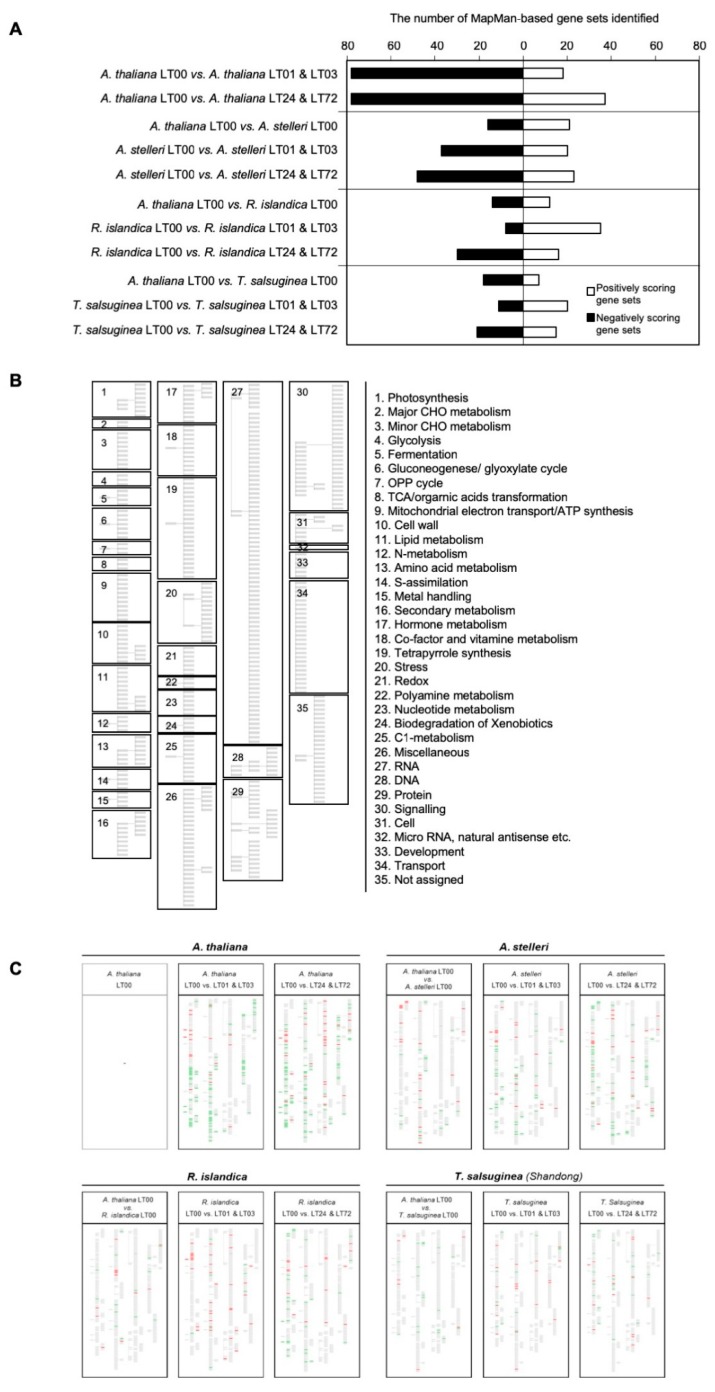
The gene sets enriched in the low-oxygen stress response of the four species. (**A**) The number of gene sets enriched in low-oxygen stress response. The gene expressions of each species under low-oxygen stress and normal conditions were analyzed by GSEA using a MapMan-based gene set database. (**B**) Graphical tree map of MapMan-based gene sets. (**C**) The positions of gene sets enriched in pairwise comparisons of *A. thaliana* and each of three closely related species on a graphical tree map of MapMan-based gene sets. For each species, gene sets enriched to low-oxygen stress at early time points (1 and 3 h) or late time points (24 and 72 h) were indicated on a graphical tree map. Red indicates positively enriched gene sets, and green indicates negatively enriched gene sets in pairwise comparisons. (**B**) is the legend for (**C**). To see the global patterns in (**C**), the group of small boxes in (B) should be virtually superimposed onto (**C**). One can compare the overall patterns of color bars in the matched boxes among the species.

Comparative analyses of gene expression between Arabidopsis and close relatives were performed using the GSEA program with a MapMan-based gene set (MM gene set) database. The analyses were independently applied to the gene expression data of species at early (LT01, LT03; LT means low-oxygen treatment) or late time (LT24, LT72) of low-oxygen treatment (Supplementary Figure S3). Gene expression data of LT08 in each species were excluded from GSEA analysis because they could not be clearly classified as early and late responses to low-oxygen treatment in *A. thaliana* and each of the closely related species (Figure 2B). With each comparison, we identified the significant gene sets in each dataset, which are summarized in Supplementary Table S4. The numbers of these gene sets in each comparison were counted to examine the degree of gene expression changes (Figure 3A). There was not much difference in the numbers of enriched gene sets between *A. thaliana* and each of the close relatives under normal growth conditions. Under low-oxygen stress, more gene sets were enriched in *A. thaliana* compared with close relatives. In addition, there were more negatively enriched gene sets (gene sets enriched with down-regulated genes) than positively enriched gene sets (gene sets enriched with up-regulated genes) in the low-oxygen stress response of each species, except for the early response to low-oxygen stress in *R. islandica* and *T. salsuginea.* To visually compare significant gene sets, gene sets enriched in each dataset were represented on a graphical tree map of MM gene sets (Figure 3B). Although several gene sets enriched in early response (LT01 and LT03) were also observed in late response (LT24 and LT72), a significantly high number of gene sets were uniquely observed in the low-oxygen responses of each species. For a better understanding of the low-oxygen stress response of each species, the significant gene sets are summarized below under the subtitle for each species.

#### 2.3.1. *A. thaliana*: Moderately Tolerant Species

More gene sets were enriched in the low-oxygen stress responses of *A. thaliana* compared with close relatives, and most of them were negatively enriched gene sets (Figure 3A). In early response, positively enriched gene sets were associated with the major energy-generating metabolic pathways, such as major CHO degradation, fermentation, gluconeogenes/glyoxylate cycle, mitochondrial electron transport/ATP synthesis, and nitrogen metabolism, whereas negatively enriched gene sets were linked to metabolic pathways that consume energy individually, such as cell wall synthesis, lipid metabolism, amino acid synthesis, nucleotide metabolism, secondary metabolism and C1 metabolism (Figure 3C and Supplementary Table S4). In contrast to early response, gene sets associated with the major energy-generating metabolic pathways, such as glycolysis, TCA/organic acids transformation and nitrogen metabolism, were negatively enriched in late response (Figure 3C and Supplementary Table S4). Growth-related gene sets, including cells and development, were also negatively enriched. As expected, enrichment of gene sets associated with fermentation, representing an anaerobic energy metabolism, suggests the transition from lactic to ethanolic fermentation in the late response to low-oxygen stress (Figure 3C and Supplementary Table S4). In conclusion, the GSEA results suggest that in *A. thaliana*, the major energy-generating metabolic pathways were upregulated at early time points and downregulated at late time points of low-oxygen stress.

#### 2.3.2. *A. stelleri*: Highly Tolerant Species

Under normal conditions, gene sets associated with photosynthesis, minor CHO metabolism, gluconeogenese/glyoxylate cycle, redox, nucleotide metabolism and transport were enriched in *A. stelleri*, while gene sets associated with mitochondrial electron transport/ATP synthesis, metal handling, stress, and protein were enriched in *A. thaliana* (Figure 3C and Supplementary Table S4).

Despite the fact that all the microarray experiments were performed with RNA from roots, photosynthesis-related gene sets including gene sets of light reaction, photorespiration and Calvin cycle were enriched in *A. stelleri* (Figure 3C and Supplementary Table S4). The photoresponses in the roots of plants have been studied [24,30]. In addition, a previous paper reported that the expression of photosynthesis-related genes was induced in *Rorippa amphibia* by submergence [15]. We also observed that root greening appeared in *A. stelleri* (Supplementary Figure S4A). Thus, the efficiency of photosynthesis was examined by measuring changes in the photosystem II photochemistry of *A. stelleri* and *A. thaliana*. The maximum quantum yields of PSII (*F*v/*F*m) in the roots of both species continuously increased during three weeks under normal growth conditions (Supplementary Figure S4B). Unexpectedly, *F*v/*F*m in the roots of three-weeks-old *A. stelleri* under normal condition was about 70% of that in leaves (Supplementary Figure S4C). These results indicated that photosynthesis also occurred in roots, although photosynthesis mainly occurred in leaves. Therefore, we carefully suspect that the high capacity of photosynthesis in the roots of *A. stelleri* may be partly in response to low-oxygen stress.

In low-oxygen conditions, positively enriched gene sets were associated with fermentation, gluconeogenesis/glyoxylate cycle, nitrogen metabolism, cofactor and vitamin metabolism, tetrapyrrole synthesis, C1 metabolism, DNA and signaling, and negatively enriched gene sets were associated with OPP pathway, TCA/organic acids transformation, mitochondrial electron transport/ATP synthesis, cell wall, lipid metabolism, amino acid metabolism, S-assimilation, secondary metabolism, abiotic stress, miscellaneous group, protein, cell, development and transport (Figure 3C and Supplementary Table S4). Many gene sets enriched in early response were also observed in late response. These results showed that the low-oxygen stress response of *A. stelleri* was continuously maintained from the early time point to the late time points. 

#### 2.3.3. *R. islandica*: Highly Tolerant Species

Under normal condition, gene sets associated with lipid metabolism, secondary metabolism, and transport were enriched in *R. islandica*, while gene sets associated with gluconeogenesis/glyoxylate cycle, cell wall, redox, nucleotide metabolism, C1 metabolism and cell were enriched in *A. thaliana* (Figure 3C and Supplementary Table S4). With respect to the numbers of enriched gene sets, there were few differences in differential gene expressions between *R. islandica* and *A. thaliana* compared with those in a pair of *A. thaliana* and *A. stelleri* or a pair of *A. thaliana* and *T. salsuginea* (Figure 3A). 

In early response, many gene sets were positively enriched, including gene sets associated with minor CHO metabolism, glycolysis, fermentation, gluconeogenesis/glyoxylate cycle, amino acid metabolism, cofactor and vitamin metabolism, biotic stress, redox, polyamine metabolism, nucleotide metabolism, biodegradation of xenobiotics, RNA, DNA, and protein (Figure 3C and Supplementary Table S4). The numbers of these positively enriched gene sets were four-fold larger than those of negatively enriched gene sets (Figure 3A). In late response, only a few gene sets enriched in early response were also observed, including gene sets associated with fermentation, gluconeogenesis/glyoxylate cycle, biodegradation of xenobiotics and RNA. Thus, overall enriched gene sets were very different between early response and late response, suggesting that *R. islandica* has unique time-dependent responses to low-oxygen stress compared with other species.

#### 2.3.4. *T. salsuginea* Shangdong: Low-Oxygen Sensitive Species

Under normal conditions, the number of gene sets enriched in *A. thaliana* was two-fold larger than those in *T. salsuginea* (Figure 3A). Gene sets associated with photosynthesis, major CHO metabolism, mitochondrial electron transport/ATP synthesis, and miscellaneous group were enriched in *T. salsuginea*, while gene sets associated with gluconeogenese/glyoxylate cycle, metal handling, auxin metabolism, tetrapyrrole synthesis, biotic stress, biodegradation of xenobiotics, C1 metabolism, transcription factor and protein were enriched in *A. thaliana* (Figure 3C and Supplementary Table S4). These results suggest that in low-oxygen conditions, *T. salsuginea* activates the energy-generating metabolic pathways, rather than defense mechanisms, which might make *T. salsuginea* very sensitive to low-oxygen stress.

In low-oxygen stress conditions, the numbers of gene sets enriched in *T. salsuginea* were fewer than those in other species (Figure 3A). In early response, positively enriched gene sets were associated with minor CHO metabolism, gluconeogenese/glyoxylate cycle, mitochondrial electron transport/ATP synthesis, metal handling, cofactor and vitamin metabolism, miscellaneous group and transport, and negatively enriched gene sets were associated with photosynthesis, OPP pathway, amino acid metabolism, and redox (Figure 3C and Supplementary Table S4). Unlike the early responses of other species, the gene set associated with fermentation was not observed in *T. salsuginea*. In late response, positively enriched gene sets were associated with gluconeogenese/glyoxlate cycle, protein and transport, and negatively enriched gene sets were associated with photosynthesis, glycolysis, OPP pathway, cell wall, amino acid metabolism, C1 metabolism, DNA, signaling and development. These results indicated that major anaerobic metabolic pathways in *T. salsuginea* such as fermentation and glycolysis were highly altered by low-oxygen stress.

### 2.4. Interspecies Variation in Reconfiguration of Energy Metabolism under Low-Oxygen Stress

Plant adaptation to anoxia always includes coping with an energy crisis. To survive the energy crisis, plant cells need to reduce their energy requirements, and also direct the limited amount of energy produced during anaerobic catabolism to the energy-consuming processes [31]. For the reason that energy metabolism is important to survive low-oxygen stress, we compared the regulation of energy metabolism in *A. thaliana*, *A. stelleri*, *R. islandica*, and *T. salsuginea* in the GSEA results. The gene sets associated with energy metabolism include glycolysis, fermentation, glyoxylate cycle, gluconeogenesis, TCA/organic acid transformation, mitochondrial electron transport/ATP synthesis, nitrogen metabolism, and photosynthesis in the MM gene set database. The enrichment of these gene sets would indicate the regulation of gene expression for the reconfiguration of energy metabolism in the low-oxygen response of each species.

**Figure 4:**
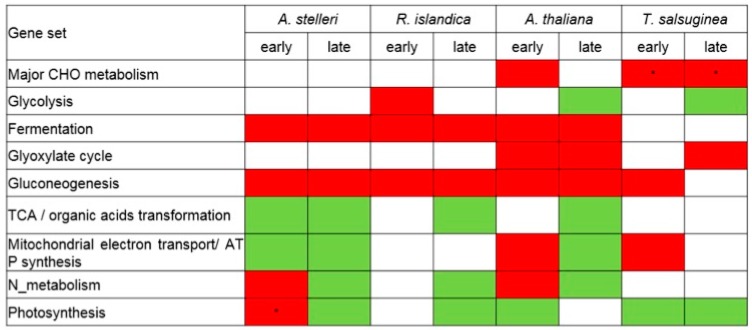
Reconfiguration of energy metabolisms in response to low-oxygen stress at the level of gene set. The regulation of energy metabolism-related gene sets was analyzed from the GSEA results of *A. thaliana*, *A. stelleri*, *R. islandica*, or *T. salsuginea*. The red box represents upregulation and green box represents downregulation. Asterisks indicate normalized levels compared to the gene expression of A. thaliana under normal conditions.

Gene sets associated with most of the energy metabolism were upregulated in early response to low-oxygen stress in *A. thaliana* (Figure 4). In late response, gene sets associated with mitochondrial electron transport/ATP synthesis and nitrogen metabolism were downregulated, while gene sets associated with fermentation, glyoxylate cycle, and gluconeogenesis were continuously upregulated. These results suggested that in Arabidopsis, energy was temporarily produced in the mitochondria via upregulation of the gene sets associated with mitochondrial electron transport/ATP synthesis and nitrogen metabolism only in early response. 

In early time points of *A. stelleri*, gene sets associated with fermentation, gluconeogenesis, nitrogen metabolism, and photosynthesis were upregulated, while gene sets associated with TCA cycle and mitochondrial electron transport/ATP synthesis were downregulated (Figure 4). The regulation of these gene sets was continuously maintained until late response except for the upregulation of gene sets associated with nitrogen metabolism and photosynthesis. Considering the enrichment of the photosynthesis-related gene set in the roots of this species compared with other species, we suspect that *A. stelleri* might additionally produce ATP through respiration, though low, using O_2_ apparently from photosynthesis under hypoxia conditions.

In early response to low-oxygen stress in *R. islandica*, gene sets associated with glycolysis and fermentation were upregulated, and gene sets associated with fermentation were continuously upregulated until late response (Figure 4). These results showed that *R. islandica* can produce more ATP through the upregulation of anaerobic energy metabolism under low-oxygen stress than other species. 

In the early response of *T. salsuginea*, although gene sets are associated with the degradation of sucrose and starch, and mitochondrial electron transport/ATP synthesis were upregulated, there were no significant changes in the regulation of gene sets associated with fermentation and glycolysis, which are the major energy-generating metabolic pathways under anaerobic conditions (Figure 4). Furthermore, glycolysis-related gene sets were downregulated in late response. These results indicated that *T. salsuginea* was sensitive to low-oxygen stress compared with other species because of a decrease in the ability to cope with energy crisis.

### 2.5. Confirmation of Changes in Root Temperatures under Low-Oxygen Stress

**Figure 5:**
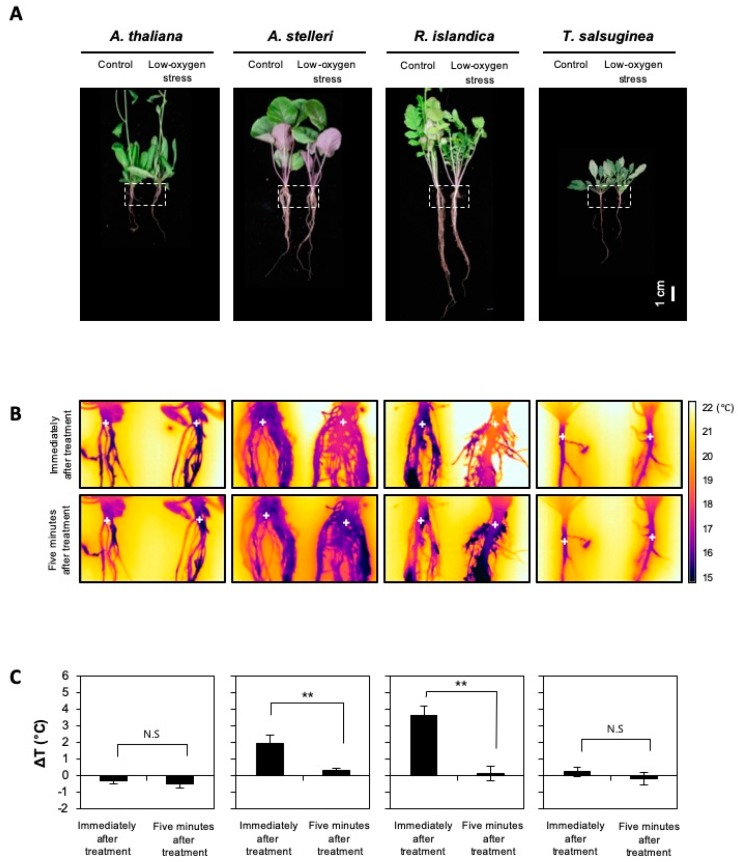
Changes in root temperature of the four species under low-oxygen stress. Five-week-old A. thaliana and closely related species were exposed to a low oxygen gas mixture (0.1% O_2_/99.9% N_2_) for 12 h. (**A**) Bright-field images of the four species. The dotted-line box indicates the position of an infrared image. (**B**) Infrared images of roots of untreated or low-oxygen-treated plants. The plus signs indicate temperature measuring positions. (**C**) Root temperatures were obtained from infrared images using ThermaCAM Quikplot software. Delta T (ΔT) is a change in temperature between the control and the low-oxygen-treated plants (ΔT = T_test_ − T_cont_). Bars represent the mean (three times) ± standard deviation. The level of statistical significance is also marked with one asterisk (*) if *p* < 0.05, two (**) if *p* < 0.01, three asterisk (***) if *p* < 0.001, and ns: not significant.

The regulation of fermentation-related gene sets representing anaerobic energy metabolism was significantly different between *A. thaliana*, *A. stelleri*, *R. islandica*, and *T. salsuginea* under low-oxygen stress (Figure 4). The biochemical process of fermentation itself generates a lot of heat [32]. It was reasonable to assume that the heat generated by the fermentation process affects temperature change in plants. Therefore, to compare the extent of fermentation induced by low-oxygen stress among the four species, root temperatures were measured using an infrared camera (Figure 5). The root temperature of *R. islandica* and *A. stelleri*, highly tolerant species, increased 3.5 °C and 1.5 °C by low-oxygen stress for 12 h, respectively, while there was no significant change in the root temperature of *A. thaliana* and *T. salsuginea* (Figure 5A–C). However, their high temperatures decreased rapidly when exposed to normal growth conditions (Figure 5C). The temperature increases after low-oxygen stress only in both high tolerant species (*R. islandica* and *A. stelleri*) implied that the heat might be generated from upregulation of fermentation activities under hypoxic conditions in these high tolerant species, as implicated by our microarray results.

## 3. Discussion

For the comparison of six species with different accessions of the Brassicaceae family based on both general growth and survival under submergence or low-oxygen treatment, we selected four representative species with different degrees of low-oxygen tolerance: *A. stelleri* and *R. islandica* as highly tolerant species, *A. thaliana* (Col-0) as a moderately tolerant species, and the Shandong ecotype of *T. salsuginea* as an intolerant species. To investigate the low-oxygen responses of these species at the molecular level, gene expression profiling using an Operon Arabidopsis microarray was carried out at various time points over 72 h under low-oxygen stress. From a comparative analysis of gene expression profiles of the four species, we concluded that the various tolerance levels of these species might be attributed to different reconfigurations of energy metabolisms under low-oxygen stress.

The low-oxygen stress tolerance levels in *A. thaliana* and close relatives were investigated to compare and identify candidates suitable for further comparative analysis of gene expression profiles. The Arabidopsis genome sequence and resources provide powerful tools that can initiate comparative genomic studies within Brassicaceae and beyond [33,34,35,36]. Cross-species hybridization is used in comparison, evolutionary and ecological studies, and for gene expression profiling of many species that lack a representative microarray platform. Recently, Arabidopsis microarrays have been successfully used in close relatives such as Brassica [37], Thellungiella [29,38] and Thlaspi [18], Rorippa [15]. 

For the comparative analysis of low-oxygen stress responses of *A. thaliana* and close relatives, we used the GSEA method, which is one of the statistical methods for analyzing gene expression profiles [39,40]. When the GSEA method was applied identically to the gene expression data of the four species, the number of enriched gene sets was much lower in close relatives than in *A. thaliana* (Figure 3). These results suggest that the change of gene expression was smaller in close relatives than in *A. thaliana*. Alternatively, it might be because the gene set used in GSEA analysis was more appropriate for *A. thaliana* gene expression studies. We ruled out the possibility of the low efficiency of cross-hybridization because the averages of hybridization signal intensities were also similar between *A. thaliana* and each of the closely related species. The MM gene set database used in GSEA analysis was constructed based on the functional categories of ~30,000 Arabidopsis genes in MapMan ontology, which was determined with information from The Arabidopsis Information Resource (TAIR), the Gene Ontology Consortium (GOC), the functionally categorized Kyoto Encyclopedia of Genes and Genomes (KEGG) database, and manually predefined keywords of TIGR release version 3.0 [41].

**Figure 6:**
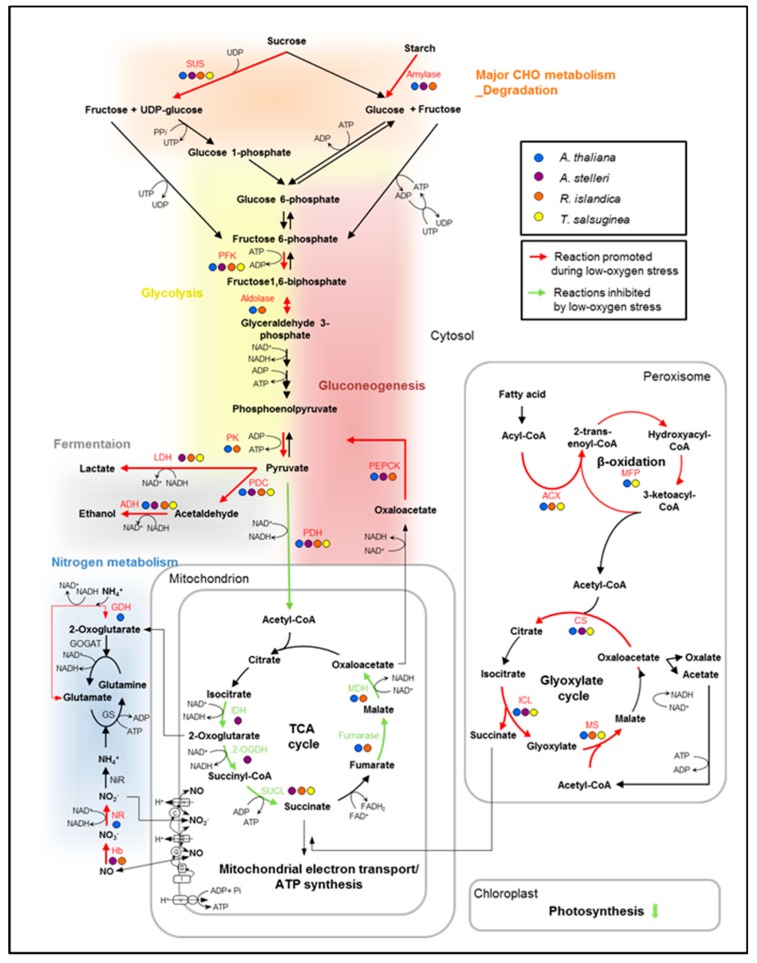
Hypothetical alterations of the metabolic pathways for ATP production in low-oxygen stress response. In the diagram, the metabolic changes caused by oxygen deprivation were characterized by comparing changes in the expression of genes that are involved in the energy-metabolic pathways. The transcript expression of key genes was compared in the low-oxygen stress response of the four species. The genes upregulated or downregulated at least two-fold in each species were indicated in the pathway. Red arrows indicate reactions that are commonly promoted during low-oxygen stress, and green arrows indicate reactions inhibited by low-oxygen stress. Abbreviations are as follows: 2-OGDH, 2-oxoglutarate dehydrogenase; ACS, acetyl-CoA synthase; ACX, acyl-CoA oxidase; ADH, alcohol dehydrogenase; CS, citrate synthase; FK, fructokinase; GDH, glutamate dehydrogenase; GOGAT, glutamine oxoglutarate aminotransferase; GS, glutamine synthase; Hb, hemoglobin; ICL, isocitrate lyase; IDH, isocitrate dehydrogenase; LDH, lactate dehydrogenase; MDH, malate dehydrogenase; MFP, multifunctional protein (in β-oxidation); MS, malate synthase; NiR, nitrite reductase; NR, nitrate reductase; PDC, pyruvate decarboxylase; PDH, pyruvate dehydrogenase; PEPCK, phosphoenolpyruvate carboxykinase; PFK, phosphofructokinase; PK, pyruvate kinase; SUCL, succinyl-CoA ligase; SUS, sucrose synthase.

In the comparative analysis of low-oxygen stress responsive gene expression in each species, the enrichment of gene sets associated with energy metabolisms was intensively examined since the balance between the consumption and production of energy is important to survive under low-oxygen stress. Consistent to our observation, an analysis with two populations of *B. rapa* with different waterlogging tolerances suggested the importance of carbohydrate supply to roots as a potential parameter for tolerance [42]. In addition, Zou et al. (2015) suggested that a tolerant variety of *Brassica napus* have a greater ability to maintain basic metabolism with lower energy consumption [43]. Thus, our results and other previous studies indicate that plants must decrease unnecessary metabolic functions to the lowest possible level under low-oxygen condition to minimize unnecessary energy consumption. We also found that each species with a different degree of low-oxygen stress tolerance had distinct strategies for reconfigurations of energy metabolic pathways. The metabolic changes caused by oxygen deprivation were investigated by comparison of changes in expression of genes associated with energy metabolic pathways including glycolysis, fermentation, glyoxylate cycle, gluconeogenesis, TCA/organic acid transformation, mitochondrial electron transport/ATP synthesis, nitrogen metabolism, and photosynthesis (Figure 6). In general, the mobilization of carbohydrates is caused by oxygen deprivation to promote substrate-level ATP production [3,4]. Amylases and sucrose synthase induced by oxygen deprivation promote the conversion of starch and sucrose to glucose. Within minutes of transfer to an oxygen-depleted environment, plant cells relying on external oxygen would limit processes that are highly energy consumptive and alter metabolism to increase the anaerobic generation of ATP by cytosolic glycolysis [44]. The glycolysis reaction is activated and the excess NADH is recycled through fermentation, regenerating NAD^+^ required to maintain glycolytic flux. When pyruvate levels increase by activation of glycolysis, the low K_m_ of mitochondrial pyruvate dehydrogenase (PDH) and high K_m_ of pyruvate decarboxylase (PDC) function to limit carbon entering the TCA cycle and promote ethanolic fermentation [7]. These reconfigurations of energy metabolism were observed in the early response of *A. stelleri* and late response of *A. thaliana*, *A. stelleri* and *R. islandica*. Previous studies with waterlogging tolerant and sensitive varieties of rapeseed showed high induction of glycolysis-related genes in both varieties of *Brassica napus* under waterlogging [43], suggesting that the upregulation of glycolysis in energy metabolism is a common response in plants regardless of their tolerance levels to waterlogging. However, our data indicated that regulations of glycolysis under low-oxygen conditions are different in each species with the different tolerance levels. Using nitrate to nitric oxide through the mitochondrial electron transport chain, plant mitochondria can drive ATP synthesis. Nitrite produced by nitrogen metabolism in cytoplasm may serve as an electron acceptor at complexes III (bc1) and IV (*COX*). This process can result in proton pumping, and can be linked to an observed ATP synthesis [45]. Nitric oxide formed in the mitochondrial electron transport process is converted to nitrate in the cytosol by hypoxia-induced hemoglobin (Hb). It is then reduced by nitrate reductase (*NiR*) to nitrite, and the cycle is repeated. Under low-oxygen stress, this reaction in nitrogen metabolism was only observed in the early response of *A. thaliana*. Fatty acids degraded by beta oxidation and glyoxylate cycle were also used as an energy source in the low-oxygen stress response of *A. thaliana* and close relatives. The glyoxylate cycle, a variation of the TCA cycle, is an anabolic metabolic pathway that occurs in the peroxisome of the plants. This cycle allows plants to take in acetate both as a carbon source and as a source of energy. Acetate is converted to acetyl CoA, and some succinate is released during the cycle. The four-carbon succinate molecule can be transformed into a variety of carbohydrates through the TCA cycle. Acetyl CoA can also react with glyoxylate to produce some NADPH from NADP^+^, which is used to drive energy synthesis in the form of ATP later in the electron transport chain [46]. A shunt to control beta oxidation and glyoxylate cycle was observed in the low-oxygen stress response of *A. thaliana* and *T. salsuginea*, which were relatively sensitive to low-oxygen stress, suggesting that low-oxygen stress-sensitive species are able to use fatty acids as an alternative source of energy in the beta-oxidation process and glyoxylate cycle during a prolonged period of low oxygen. Recently, the glyoxylate cycle was also reported as having unique flexibility in energy metabolism in mycobacteria under oxygen-limiting condition [47]. 

At the whole-plant level, complete submergence leads to a dramatic shift in the carbon budget and energy status, potentially resulting in death. Underwater photosynthesis provides some relief for this problem, with the leaves still submerged, [48], but underwater photosynthesis can be limited by low light and CO_2_ availability. When plants are facing low-oxygen stress, photosynthesis is reduced as a result of stomatal closure, decreased activity of carboxylation enzymes and decreased leaf chlorophyll content [49]. Photosynthesis was inhibited by low-oxygen stress in *A. thaliana*, *R. islandica* and *T. salsuginea* as conjectured, but not in *A. stelleri*. In order to further examine the efficiency of photosynthesis of *A. stelleri* during low-oxygen stress, the maximum quantum yield of PSII (Fv/Fm) of *A. stelleri* was measured after low-oxygen stress (Supplementary Figure S4). After 72 h of low-oxygen treatment, the Fv/Fm values of the roots and leaves of *A. stelleri* decreased by 51% and 25%, while this value for the roots and leaves of *A. thaliana* decreased by 100% and 60%, respectively. The results indicated that *A. stelleri* was allowed to retain some capacity of PSII photochemistry during low-oxygen stress. In spite of the root samples, the expression of photosynthesis-related genes and the efficiency of photosynthesis were changed by oxygen deprivation. It is not clear what caused the induction of photosynthesis-related genes in *A. stelleri* after low-oxygen treatment. Sasidharan et al. (2013) speculated that sugar starvation might be one of the causes of these gene induction [15]. However, we did not observe the significant differences between an expression of sugar starvation related genes in *A. stelleri* and other species. Plants sense ambient light conditions and modulate their developmental processes by utilizing multiple photoreceptors such as phytochromes, cryptochromes, and phototropins. Even roots, which are normally not exposed to light, express photoreceptors and can respond to light by developing chloroplasts [15,30]. Roots show various photoresponses, and light influences many aspects of root development including root extension, geosensitivity, and lateral root formation [24]. 

In conclusion, this study showed that each species with a different degree of low-oxygen stress tolerance distinctively reconfigures energy metabolic pathways under low-oxygen stress. The comparison of the differences between the responses of the four species to a particular stress helped to explain their ability to withstand stress. In *A. thaliana*, the dynamical reconfiguration of energy metabolisms in early response was restricted in late response to low-oxygen stress, suggesting that the survival of *A. thaliana* is seriously affected when exposed to a low-oxygen condition for a prolonged period. Given the fact that photosynthesis genes were enriched in *A. stelleri*, it is tempting to speculate that in low-oxygen stress conditions, this highly tolerant species sustains some ATP levels through respiration using O_2_, presumably from high photosynthesis capacity. Therefore, this early response of *A. stelleri* might lead to a better chance of survival under low-oxygen stress. However, it should be noted that our speculation is still hypothetical and it still requires further study. *R. islandica*, one of the highly tolerant species, retained some energy via ATP produced by anaerobic energy metabolism during a prolonged period of low oxygen. In *T. salsuginea*, a relatively intolerant species, there were no significant changes in the expression of genes involved in anaerobic energy metabolisms, suggesting that their ability to cope with energy crises under low-oxygen stress decreased. The comparative analysis of species with different degrees of low-oxygen stress tolerance provides a more comprehensive understanding of the reconfiguration of energy metabolism in the low-oxygen response of plants. Furthermore, the outcome of this study will help to develop flood-resistant or flood-tolerant crop plants.

## 4. Materials and Methods 

### 4.1. Plant Materials and Growth

Seeds of *A. thaliana* (Columbia, Landsberg erecta and Wassilewskija), *T. salsuginea* (Shandong and Yukon), and *T. parvula* were obtained from the Arabidopsis Biological Resource Center at Ohio State University (Columbus, OH, USA), and the seeds of *T. arvense* were obtained from B&T World Seeds (http://b-and-t-world-seeds.com/). The seeds of Arabidopsis mutant *eskimo 1* (*esk-1*) were provided by Dr. John Browse at Washington State University (USA) and the seeds of *R. islandica* were provided by Dr. Hong-keun Choi at Ajou University (Korea). The seeds of wild-type *A. stelleri* var. *japonica* were collected from the seaside near the city of Pohang, Korea. 

For submergence treatment, the seeds were sown in Sunshine #5 soil (Sun Gro Horticulture, Canada) in pots. All the plants were grown at 23 °C in long-day conditions (16 h light/8 h dark) with approximately 100 µmol m^−2^ s^−1^ light intensity in an environmentally controlled growth chamber. For low-oxygen treatment and gene expression experiments, the seeds were surface-sterilized, imbibed in water in darkness at 4 °C for 3 days for synchronized germination, and grown in growth medium (Murashige and Skoog half-strength solution containing 1% phytoagar).

### 4.2. Submergence and Low-Oxygen Treatment 

For submergence treatment, 13~15 plants were grown in a pot until the developmental stage immediately before bolting in soil. Three-week-old Arabidopsis or four-week-old closely related species were entirely submerged in a tank of water 40 cm deep and held for 7 days at 23 °C in a dark condition.

For low-oxygen treatment, 18~20 plants were grown on an agar plate until the stage of emergence of three to four leaves. Two-week-old Arabidopsis or three-week-old closely related species were transferred to an airtight vacuum chamber in an environmentally controlled growth cabinet. A low oxygen gas mixture (0.1% O_2_/99.9% N_2_) was supplied continuously to the airtight vacuum chamber to retain low O_2_ conditions during treatment. The oxygen concentration in the airtight vacuum chamber was monitored by an XP-3180 oxygen meter (Cosmos, Osaka, Japan). This treatment was carried out at 23 °C for 5 days in a dark condition. In each experiment, plant survival was scored based on the survival of shoot apical meristem after recovery.

Each experiment for submergence and low-oxygen treatment was repeated three times.

### 4.3. RNA Isolation and Purification

Total RNA was isolated using an RNeasy Plant Mini Kit (Qiagen, Hilden, Germany), following the manufacturer’s instructions. Briefly, the root samples of 18~20 plants on an agar plate were homogenized in the presence of liquid nitrogen, and were lysed in a buffer containing guanidine isothiocyanate (GITC). The lysed samples were placed in the RNeasy columns and washed with an ethanol-containing buffer. Total RNA was eluted with RNase-free water. For ethanol precipitation, 1 mL of 95% ethanol and one-tenth volume of 3 M NaOAc, pH 5.2 were added and the total RNA samples were held at −80 °C for 20 min. After centrifuging at 12,000× *g* for 15 min at 4 °C, the RNA pellets were washed with 1 mL of 70% ethanol and centrifuged at 12,000× *g* for 5 min at 4 °C. The pellets were then dissolved in RNase-free water. The concentration and purity of this isolated total RNA were determined by measuring the absorbances at 260 nm and 280 nm.

### 4.4. Microarray Experiment, Image Acquisition, Data Acquisition and Normalization

For the microarray experiment, plants were grown in an environmentally controlled growth chamber at 23 °C under constant light (approximately 100 µmol m^−2^s^−1^). Total RNA (5 μg) from root samples of *A. thaliana* (Col-0), *A. stelleri*, *R. islandica*, and *T. salsuginea* (Shandong) exposed to low oxygen for 0, 1, 3, 8, 24, or 72 h was reverse transcribed using reverse transcription (RT) primer tagged with either Cy3-3DNA or Cy5-3DNA capture sequence of Array 900 MPX Expression Array Detection Kits (Genisphere, Hatfield, PA, USA). The synthesized cDNA including capture sequence were fluorescently labeled by Cy3-3DNA or Cy5-3DNA based on the sequence complementary to the 3DNA capture reagent, which contained an average of 900 fluorescent dyes. The labeled cDNA was hybridized on an Operon Arabidopsis Version 3.0 microarray consisting of 26,173 probes spotted with synthetic 70-mer oligonucleotides on aminosilane-coated slides by the David Galbraith lab, University of Arizona. The hybridization and washing procedures were performed according to Genisphere technical protocol. After washing, the slides were immediately scanned using an ArrayWoRx (Applied Precision, Issaquah, WA, USA). To maximize the camera’s dynamic range without saturation and to normalize the two channels for signal intensity, the exposure setting was adjusted so that the intensity level of the brightest spot on a slide was 80 to 90%. Experiments were performed a replicated dye swap for each microarray (Supplementary Figure S3).

Intensity values were quantified from the pairs of TIFF image files from each channel using version 5.6 ImaGene software (BioDiscovery, Los Angeles, CA, USA). Analyses were done using the version 4.1 GeneSight software package (BioDiscovery, Los Angeles, CA, USA). For each slide, the local background was subtracted from the signal intensity, and the minimum intensity was raised to 20 by using the “floor” function. The mean intensity for each element was normalized by the locally weighted scatterplot smoothing (LOWESS) method and expression values (log_2_) were calculated by comparing intensities from (1) each three species and Arabidopsis before treatment (LT00) (“comparison each three species versus *A. thaliana* as reference” in Supplementary Table S2) or (2) each species at each time points (LT01, 03, 08, 24, and 72) and each species before treatment (“comparison low-oxygen versus control” in Supplementary Table S2) using the GeneSight software. A two-sided t-test was performed using the R Statistical Package [50], to determine which genes were significantly differentially expressed between the low-oxygen treated and control groups, and Benjamini–Hochberg false discovery rate (FDR) multiple testing correction (https://cran.r-project.org/web/packages/ simulator/vignettes/fdr.html) and alpha level of 0.05 was applied [51]. The FDR method was used for testing and adjustment of p-values.

The significantly differentially expressed genes were identified using the following parameters as a confidence threshold: adjusted *p* value < 0.05 and log_2_ fold change ≤ −1.0 or ≥ 1.0.

### 4.5. GSEA Analysis Using MapMan-Based Gene Set Database

A MapMan-based gene set (MM gene set) database was constructed based on functional categories of MapMan ontology to analyze microarray data of Brassicaceae species at the gene set level using Gene Set Enrichment Analysis (GSEA) software. MapMan ontology (file name: Map files/Ath_AGI_TAIR9.txt) was downloaded from the MapMan website (http://mapman.gabipd.org/web/guest/mapman-store), where each of the genes was assigned to the corresponding categories of a tree-like hierarchical structure classified according to the functional categories. Each gene set was defined by the group of genes in each of the hierarchically functional categories assigned by a BIN code of MapMan ontology. “MM gene set_type I” was constructed by genes grouped in level 1 of the BIN code of hierarchically functional categories. However, the number of genes included in a few categories of level 1 was too small or too large to calculate the statistical significance using GSEA software. Hence, “MM gene set_type II” was additionally constructed by genes grouped in levels 2 or 3 of the BIN code of these categories. Each type of functional gene set database was separately carried out on GSEA software but all GSEA results obtained by “MM gene set_type I” and “MM gene set_type II” were used for analysis. A graphical tree map of MapMan-based gene sets was drawn based on the hierarchical structure of functional categories in MapMan ontology using version 1.1 Scalable Vector Graphics (SVG) software (http://www.w3.org/Graphics/SVG/) (Figure 3B).

The GSEA was performed using version 2.0.7 of the GSEA-P software downloaded from the GSEA website (http://www.broadinstitute.org/gsea/) [40]. To calculate the significance of the enrichment score (ES), class labels were randomly permuted and ES were recalculated 1000 times. In this study, the cutoff for significance of ES was defined as the score according to a *P* value of 0.05 and an FDR value of 0.25. A statistically significant value for the gene sets represented by less than 10 genes was defined to be a *P* value of 0.1 since a small population size has a negative influence on statistical significance. GSEA evaluated a query microarray data set by using the MapMan-based gene set (MM gene set) database.

### 4.6. Infrared Thermography

*A. thaliana* (Col-0), *A. stelleri*, *R. islandica*, and *T. salsuginea* (Shandong) were grown in soil under normal conditions for 5 weeks to take infrared images of its root surface. To investigate the root temperature changes by low-oxygen stress, plants were exposed to a low oxygen gas mixture (0.1% O_2_/99.9% N_2_) for 12 h and the root temperature was measured immediately and 5 min after low-oxygen stress. The soil was quickly removed from roots of untreated or low-oxygen-treated plants before measuring the temperature of roots. The root temperature was measured from infrared images captured using ThermaCAM T355 infrared camera (FLIR Systems, Wilsonville, OR, USA), which are devices capable of sensing this radiation in the form of infrared light. The images were analyzed using ThermaCAM Quikplot software (FLIR Systems, Wilsonville, OR, USA). Root temperature measured from infrared images was compared between the control and the low-oxygen treated plants (ΔT = Ttest − Tcont). Infrared thermography measured root temperature with three different biological samples.

### 4.7. ITS PCR Amplification and Analysis

Genomic DNA was isolated from 1 g of fresh leaves using a genomic DNA extraction kit (RBC; Real Biotech Corporation, Taiwan). The genomic DNA was then used as a template for the PCR amplification of ITS using the two universal ITS primers described by White et al. (1990) [52]. The ITS5 primer, 5′-TCCTCCGCTTAT TGATATGC-3′, was derived from the 3′ end of 18S rRNA, and the ITS4 primer, 5′-GGAAGTAAAAGTCGTAACAAGG-3′, was derived from the 5′ end of 25S rRNA. The ITS fragments of each species were amplified in a 20-μL reaction volume containing 2 μL of 10× PCR buffer, 2.5 mM each dNTPs, about 50 ng of genomic DNA, one unit of *Taq* DNA polymerase (Takara EX, Shiga, Japan), 8 pmole primers in 30 cycles of denaturation at 94 °C for 30 s, annealing at 55 °C for 30 s, and extension at 72 °C for 30 s. After the PCR reaction, 5 μL of samples were analyzed on 1.4% agarose gel. The amplified ITS fragments were sequenced (Cosmogenetech co, Korea). The ITS DNA sequences were compared using the cluster algorithm of AliBee (http://www.genebee.msu.su/services/malign_reduced.html).

### 4.8. Semi-Quantitative RT-PCR

Total RNA (2 μg) was extracted from the roots of 18~20 plants exposed to low oxygen for 0, 1, 3, 8, 24, or 72 h. Residual DNA was removed by treatment with DNase I (1U/ mg RNA)(Promega, Madison, WI, USA) for 30 min at 37 °C, and then DNase I was inactivated by incubation for 5 min at 72 °C. The RNAs were reverse-transcribed with Superscript^®^II Reverse transcriptase (Invitrogen, Carlsbad, CA, USA). The target cDNA was PCR-amplified in a 20 μL reaction volume containing 2 μL of 10× PCR buffer, 25 mM of each dNTP, 1 μL of cDNA, and one unit of *Taq* DNA polymerase (Takara, Shiga, Japan), plus 8 pmol of the appropriate primer sets (see Supplementary Table S5) in 25 cycles of denaturation at 94 °C for 30 s, annealing at 55 °C for 30 s, and extension at 72 °C for 30 s. The amplified PCR products of the target gene were evaluated by 1.5% agarose gel electrophoresis in TBE buffer stained with ethidium bromide (Sigma-Aldrich, St. Louis, MO, USA). Semi-quantitative RT-PCR was repeated with three different biological samples and the representative results were presented. Gene expression level of RT-PCR result was measured from signal intensities on photograph of an agarose gel and normalized using by an internal control such as TUB2 and Actin2. The Pearson correlation coefficients (R^2^) were calculated between the expression level of target genes in RT-PCR and microarray data.

### 4.9. Measurement of Photosynthetic Activity in Root 

The maximum quantum yield of PSII (*F*v/*F*m) was measured in the roots of each species for comparison of photosynthetic activity in *A. thaliana* (Col-0) and *A. stelleri*. For measurement of *F*v/*F*m under normal growth conditions, *A. thaliana* (Col-0) and *A. stelleri* were grown on agar media in long-day conditions (16 h light/8 h dark) or darkness for 1, 2, or 3 weeks. For comparison of the effect of low-oxygen stress on *F*v/*F*m, two-week-old *A. thaliana* (Col-0) and three-week-old *A. stelleri* grown on agar plates were exposed to low oxygen (0.1% O_2_/99.9% N_2_) for 72 h. Before *F*v/*F*m measurement, all plants were dark adapted for 30 min. The *F*v/*F*m was measured using a hand-held fluorometer FluorPen FP100 (Photon Systems Instruments, Czech Republic). Each measurement was repeated three times with ten replications per experiment.

## Figures and Tables

**Table 1:** Summary of effects of low-oxygen stress on Arabidopsis and closely related species. The plus sign indicates degree of resistance to each treatment: +++, high resistance; ++, medium resistance; +, low resistance.

Treatment	Physiological Characters Affecting Tolerance	Species with Different Accessions									
		*A. thaliana* (Col-0)	*A. thaliana* (esk-1)	*A. thaliana* (Ws)	*A. thaliana* (Ler)	*A. stelleri*	*R. islandica*	*T. arvense*	*T. salsuginea* (Shandong)	*T. salsuginea* (Yukon)	*T. parvula*
Submergence treatment	Growth	++	+	++	++	++	+++	+	+	+	+
	Survival	++	+++	+++	+++	+++	+++	+	+	++	+
Low-oxygen treatment	Growth	++	++	+	+	+++	+++	++	+	++	+
	Survival	+	++	+	+	++	+++	+	++	+	+
Tolerance range to low-oxygen stress		Moderate				High		Low			

## Data Availability

The microarray data used in this research are available in the Gene Expression Omnibus (GEO) repository under accession numbers of GSE129468. (https://www.ncbi.nlm.nih.gov/geo/query/acc.cgi?acc=GSE129468).

## Supplementary Materials

Supplementary materials can be found at https://www.mdpi.com/1422-0067/21/5/1787/s1.
