# Supplementary material for: Modulation of Energy Metabolism Is Important for Low-Oxygen Stress Adaptation in Brassicaceae Species
Source: Int J Mol Sci. 2020 Mar 5;21(5):1787. doi: 10.3390/ijms21051787 (PMC7084654; doi:10.3390/ijms21051787)
Supplement: Supplementary file 1 [file ijms-21-01787-s001.zip › supplementary figures.pdf]

International Journal of
Molecular Sciences

MDPI

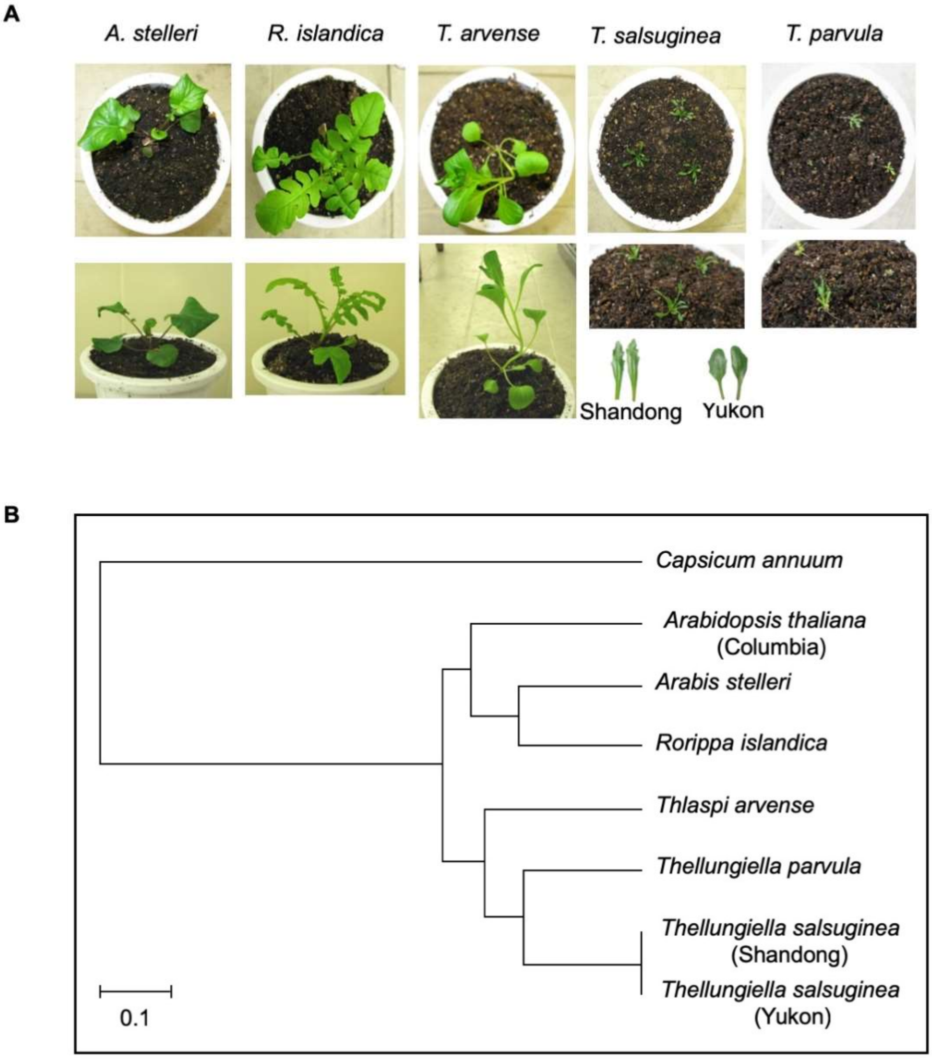

Figure S1. Morphologic and phylogenetic relationships of closely related species. (A) Morphology of four-week-old closely related species. (B) Phylogenetic tree of A. thaliana and closely related species based on nuclear internal transcribed spacer (ITS) sequences. The phylogenetic tree of ITS of *A. thaliana* and closely related species was obtained by the cluster algorithm of AliBee (http://www.genebee.msu.su/services/malign_ reduced.html). Capsicum annuum, a family of Solanaceae, was used as an outgroup.

Species	Name	Gene ID	Microarray analysis Relative expression level (log_2)					RT-PCR analysis	R^2 value
			LT01	LT03	LT08	LT24	LT72	LT00 LT01 LT03 LT08 LT24 LT72	
A. thaliana	Alcohol dehydrogenase (ADH)	At1g77120	1.31	2.10	2.36	1.69	0.30		0.8
	Pyruvate decarboxylase 1 (PDC1)	At4g33070	1.98	1.66	1.29	0.28	-1.22		0.9
	Pyruvate decarboxylase 2 (PDC2)	At5g54960	1.82	1.80	1.72	1.08	-0.08		0.8
	Sucrose synthase 1 (SUS1)	At5g20830	2.03	2.54	2.82	2.96	0.92		0.5
	Ethylene response factor (ERF)	At5g61600	1.04	1.16	1.03	1.49	1.92		0.7
	Ethylene response protein 2 (ETR2)	At3g23150	1.87	1.52	0.92	0.95	1.41		0.8
	Heat shock protein (HSP17.6A)	At1g59860	5.27	5.22	4.97	4.53	-0.20		0.6
	Tubulin beta-2 chain (TUB2)	At5g62690	-0.14	-0.19	-0.49	-0.56	-0.29		0.9
R. Islandica	Alcohol dehydrogenase (ADH)	At1g77120	2.90	4.13	3.59	2.72	2.18		0.8
	Pyruvate decarboxylase 1 (PDC1)	At4g33070	3.08	4.90	4.38	4.44	2.27		0.6
	Pyruvate decarboxylase 2 (PDC2)	At5g54960	3.55	3.71	3.66	3.43	2.94		0.9
	Ethylene response protein 2 (ETR2)	At3g23150	0.26	0.45	0.74	0.32	0.18		0.7
	Ethylene response factor (ERF)	At5g61600	0.06	0.45	0.92	0.36	-0.07		0.5
	Tubulin beta-2 chain (TUB2)	At5g62690	-0.18	-0.19	-0.42	-0.54	-0.24		1.0
A. stelleri	Alcohol dehydrogenase (ADH)	At1g77120	2.05	3.88	3.80	2.82	1.78		0.9
	Pyruvate decarboxylase 2 (PDC2)	At5g54960	2.71	2.81	2.63	2.47	0.95		0.9
	Tubulin beta-2 chain (TUB2)	At5g62690	-0.09	-0.36	-0.06	-0.84	-0.09		1.0
T. salsuginea	Pyruvate decarboxylase 2 (PDC2)	At5g54960	0.85	1.69	1.51	0.98	0.54		0.8
	Long-chain acyl-CoA synthetase 4 (LACS4)	At4g23850	-0.07	-0.02	0.10	-0.98	0.07		0.7
	protein phosphatase 2C (ABI1)*	At4g26080	0.79	0.05	0.28	0.32	1.28		0.8
	40S ribosomal protein S25 (RPS25A)*	At2g16360	-0.76	0.00	-0.80	-1.95	-2.47		0.9
	Calmodulin-domain protein kinase 9 (CPK9)*	At3g20410	0.28	-0.04	0.17	-4.21	-3.60		0.6
	Actin2*	At3g18780	0.00	-0.04	0.20	0.00	0.32		1.0

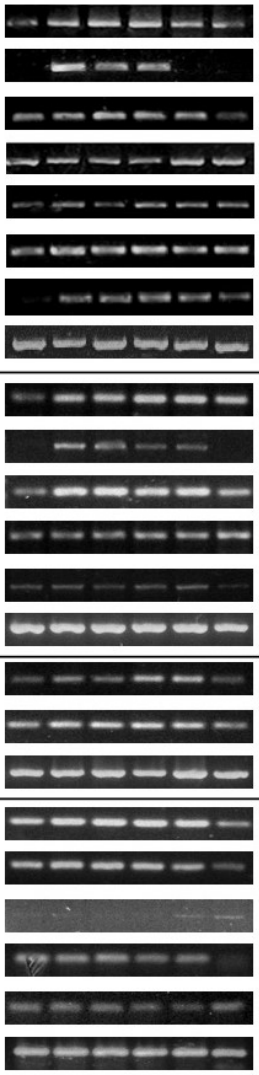

Figure S2. Verification of microarray data by RT-PCR. RT-PCR analysis was performed with low-oxygen responsive gene transcripts from two-week-old *A. thaliana* and three-week-old closely related species after low-oxygen treatment for six different durations. TUB2 or ACT in each species was used as an internal control. The values in the far right column indicate correlation coefficient (R^2) with the expression level of target genes in microarray data. Asterisks indicate the use of primers designed on cDNA sequences of *T. salsuginea*. The remaining PCR primers were based on *A. thaliana* sequence because of a lack of sequence information on *R. islandica* and *A. stelleri*.

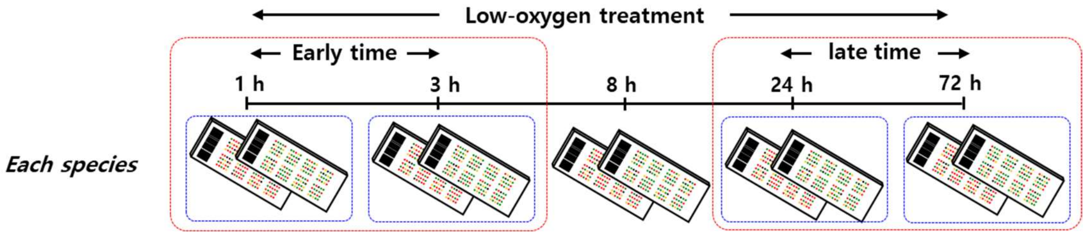

Figure S3. The replicates for GSEA analysis and microarray experiments. All experiments were performed with a replicated dye swap at 5 time points for each microarray. Blue boxes (each time point of microarray experiment) mean technical replicates of dye swap replicates within the same treatment and a time point. Red boxes (group of early- and late-time for GSEA analysis) indicate biological replicates with the samples derived from another time point (i. e. 1 and 3 hours for early time).

International Journal of
Molecular Sciences

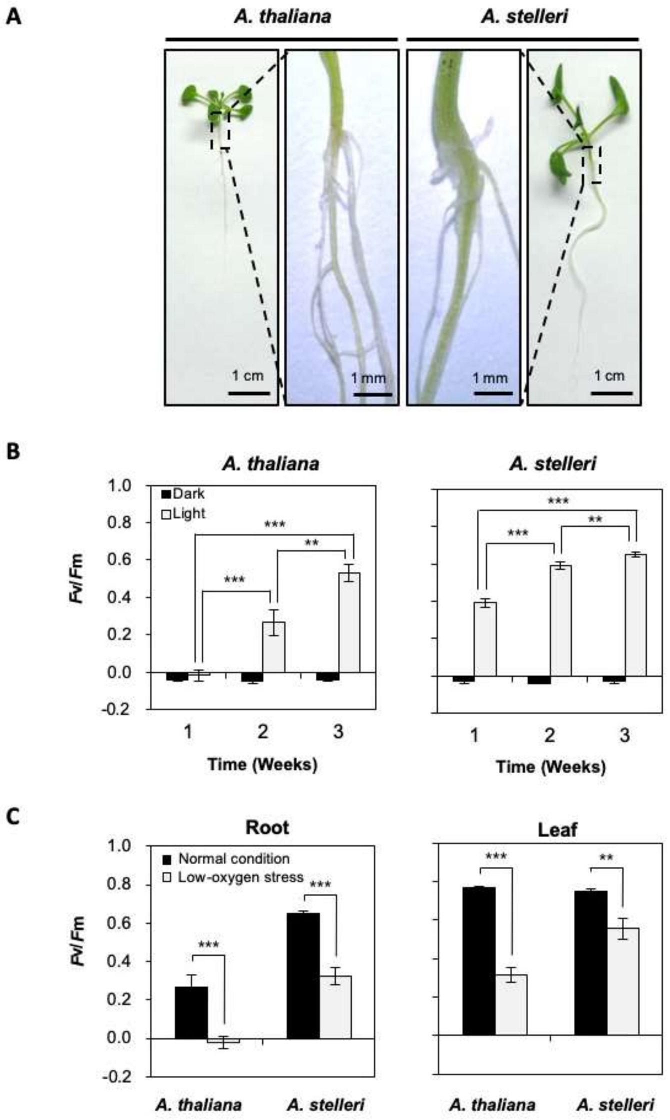

Figure S4. Maximum quantum yield (*F*v/*F*m) of photosystem II in *A. thaliana* and *A. stelleri.* **(A)** Root greening of two-week-old *A. thaliana* and *A. stelleri*. (**B)** *F*v/*F*m in the roots of *A. thaliana* and *A. stelleri* at similar developmental stages. **(C)** *F*v/*F*m in the roots and leaves of *A. thaliana* and *A. stelleri* exposed to low-oxygen stress (0.1% O_2/99.9% N_2) for 72 h. Bars represent the mean (three times) ± standard deviation (n=10). The level of statistical significance is also marked with one asterisk (*) if $p < 0.05$, two (**) if $p < 0.01$ and three asterisk (***) if $p < 0.001$.
